# The Annual Wellness Visit Health Risk Assessment: Potential of Patient Portal-Based Completion and Patient-Oriented Education and Support

**DOI:** 10.1093/geroni/igae023

**Published:** 2024-02-29

**Authors:** Danielle S Powell, Mingche M J Wu, Stephanie Nothelle, Kelly Gleason, Esther Oh, Hillary D Lum, Nicholas S Reed, Jennifer L Wolff

**Affiliations:** Department of Hearing and Speech Sciences, University of Maryland, College Park, Maryland, USA; Department of Health Policy and Management, Johns Hopkins Bloomberg School of Public Health, Baltimore, Maryland, USA; Department of Health Policy and Management, Johns Hopkins Bloomberg School of Public Health, Baltimore, Maryland, USA; Division of Geriatric Medicine and Gerontology, Johns Hopkins University School of Medicine, Baltimore, Maryland, USA; School of Nursing, Johns Hopkins University, Baltimore, Maryland, USA; Division of Geriatric Medicine and Gerontology, Johns Hopkins University School of Medicine, Baltimore, Maryland, USA; Division of Geriatric Medicine, Department of Medicine, University of Colorado School of Medicine, Aurora, Colorado, USA; Department of Epidemiology, Johns Hopkins Bloomberg School of Public Health, Baltimore, Maryland, USA; Department of Health Policy and Management, Johns Hopkins Bloomberg School of Public Health, Baltimore, Maryland, USA

**Keywords:** Consumer health information technology, Health services, Medicare, Preventive care

## Abstract

**Background and Objectives:**

Patient portals are secure online platforms that allow patients to perform electronic health management tasks and engage in bidirectional information exchange with their care team. Some health systems administer Medicare Annual Wellness Visit (AWV) health risk assessments through the patient portal. Scalable opportunities from portal-based administration of risk assessments are not well understood. Our objective is 2-fold—to understand who receives vs misses an AWV and health risk assessment and explore who might be missed with portal-based administration.

**Research Design and Methods:**

This is an observational study of electronic medical record and patient portal data (10/03/2021–10/02/2022) for 12 756 primary care patients 66+ years from a large academic health system.

**Results:**

Two-thirds (*n* = 8420) of older primary care patients incurred an AWV; 81.0% of whom were active portal users. Older adults who were active portal users were more likely to incur AWV than those who were not, though portal use was high in both groups (81.0% with AWV vs 76.8% without; *p* < .001). Frequently affirmative health risk assessment categories included falls/balance concerns (44.2%), lack of a documented advanced directive (42.3%), sedentary behaviors (39.9%), and incontinence (35.1%). Mean number of portal messages over the 12-month observation period varied from 7.2 among older adults affirmative responses to concerns about safety at home to 13.8 for older adults who reported difficulty completing activities of daily living. Portal messaging varied more than 2-fold across affirmative health risk categories and were marginally higher with greater number affirmative (mean = 13.8 messages/year no risks; 19.6 messages/year 10+ risks).

**Discussion and Implications:**

Most older adults were active portal users—a group more likely to have incurred a billed AWV. Efforts to integrate AWV risk assessments in the patient portal may streamline administration and scalability for dissemination of tailored electronically mediated preventive care but must attend to equity issues.

**Translational Significance:** Health system adoption of the Medicare Annual Wellness Visit (AWV), although slow to take hold, has been increasing—with attention to integration of the visit in systematic initiatives like the patient portal. This study informs who receives an AWV and who may be missed by potential future embedded facilitation of the AWV or educational prevention components through a patient portal. Our work identifies specific health risks which may be particularly beneficial for patient portal-based initiatives, identifies patient health risk concerns which currently facilitate a greater volume of patient-initiated portal messages, and where attention to health equity is important for prevention.

Identifying and scaling strategies that elicit and integrate the patient and family voice in communication and care delivery have been a sustained priority in national policy efforts (1). Electronic medical record (EMR) systems with a patient-facing component are a powerful platform for engaging patients in care (2,3). The patient portal is a secure online platform connected to the EMR that may be used to facilitate access to information about the patient’s health (4–7), including the collection of patient generated health information (8–11). The integration of the patient voice in care may have particular benefit for older adults with complex health needs, for whom the formulation of a visit agenda may be more difficult (12).

The Medicare Annual Wellness Visit (AWV) is a preventive care visit that creates a framework for collecting patient-reported information (13–15). Established in 2011 under the Patient Protection and Affordable Care Act (16,17), the AWV affords Medicare reimbursement for clinicians to engage in preventive health conversations during a visit that is distinct from a physical exam or problem-focused visit (14). Required components of the AWV include a health risk assessment (17), medication reconciliation, review of medical and family history, and evaluation of risk factors through the health risk assessment. Recent studies indicate that having a billed AWV is associated with increases in preventive health behaviors and improved care such as vaccinations, cancer screening, and dementia diagnosis (18–20). However, previous studies that have examined AWV completion have defined visits using billing codes, without examining how specific health risk concerns are reported (15).

Integrating delivery of the AWV into clinical practice is time intensive and although increasing (21), remains underutilized in many organizations (21–23). The patient portal has the potential to streamline AWV administration and reduce staff time by electronically sending the health risk assessment for the patient to self-administer in advance of the visit (24–26). In addition, AWV response integration within the portal may facilitate proactive care by delivering portal-based education or referrals tailored to address identified risks (26). Using EMR and patient portal data from a large academic health system our objective is 2-fold—to understand characteristics of older adults who do (vs do not) receive an AWV and health risk assessment, and in doing so, those patients who might be missed with portal-based administration of the AWV health risk assessment.

## Method

### Study Sample

This study draws on patient-level health and demographic characteristics from the EMR for a sample of adults aged 65 years and older who were established primary care patients at a large academic health system. Eligibility criteria included 2 or more primary care visits within a 2-year period between 2017 and 2022, having incurred at least one encounter during the observation period (October 3, 2021–October 2, 2022), and being enrolled in Medicare and eligible for the AWV based on being 66 years or older (beneficiaries are ineligible during the first 12 months of enrollment) as previously described (27). A billed AWV was based on current procedural terminology codes G0438 or G0439 (14,24). Our final analytic sample comprises older adults who answered ≥1 health risk assessment question to distinguish AWV billed visits, which ensured incorporation of at least one component of the health risk assessment into visit practice in alignment with our study objectives. This study was approved by the Johns Hopkins School of Medicine Institutional Review Board.

### Annual Wellness Visit Health Risk Assessment

The AWV health risk assessment at our health system includes more than 30 items (see Supplementary Table 1). At our health system, health risk assessment items are most often administered by medical assistants and are recorded within an EPIC flowsheet by question. As the specific item wording changed during our observation period, we harmonized items into 28 health risk assessment categories. These categories were grouped into 2 domains following the patient-reported outcomes measurement information system (PROMIS) (28), 17 were categorized as mental health risk categories and 11 were categorized as physical health risk categories. Health risk assessments were linked to billed AWV encounters when within 30 days of the billed AWV, when they were outside 330 days of another billed AWV, and when one or more health risk category was complete. An assessment was defined as completed if a response for all 28 categories was recorded. We note affirmative responses (yes/no) for each health risk category and the time stamp of the assessment entry in the EMR.

### Patient Portal Use

We constructed metrics of patient portal use as previously described (29). We defined an *active portal user* as having a registered portal account and having logged in to complete at least one activity (eg, view lab results, view chart, message a clinician) during the observation period. A portal *episode* was defined as having logged in and completed at least 1 other portal activity. Portal messaging refers to the volume of patient- or care partner-initiated messages sent through the patient portal requesting medical advice.

### Covariates

Patient demographic characteristics include age in years, sex (female/male), race (White/Black/Other), English as preferred language (yes/no), and married (yes/no). From the EMR problem list and encounter International Classification of Disease (ICD) codes (30,31) present at the start of the observation period, we identify dementia, diabetes, hearing loss, chronic obstructive pulmonary disease, chronic kidney disease, rheumatoid arthritis or osteoarthritis, and hypertension.

### Statistical Analysis

We first examine differences in sample characteristics by billed AWV status, with statistical significance assessed based on chi squared, Kruskal–Wallis test, or *t*-test as appropriate. We then restrict our sample to older adults with an AWV and at least 1 completed health risk assessment category to comparatively evaluate the frequency of risk assessment category completion and affirmative responses by portal user status as well as absolute between-group differences by portal use. Lastly, we evaluate the volume of patient-initiated portal messages by type and number of affirmatively responded health risk assessment categories.

## Results

### Characteristics of the Study Sample by Annual Wellness Visit and Portal Use

Of 12 756 older adults eligible for a Medicare Annual Wellness Visit, 8420 (66.0%) incurred a billed AWV and completed questions for ≥1 health risk assessment category during the observation period. The 12 756 primary care patients who were eligible for the AWV had a mean age of 76.2 years, were predominantly female, White, and indicated English as the preferred language (Table 1).

**Table 1.** Characteristics of Eligible Older Adults by Billed Annual Wellness Visit (AWV).

Characteristic	Overall	AWV billed		p Value^c^
	*n* = 12,756	Yes *n* = 8420	No *n* = 4336	
Age in years, mean (*SD*)	76.2 (7.7)	76.0 (8.3)	76.6 (7.4)	**<0.001**
Female sex, *n* (%)	7,857 (61.6)	5,187 (61.6)	2,670 (61.6)	0.98
Race, *n* (%)				**<0.001**
White	8,434 (67.3)	5,792 (70.0)	2,642 (62.0)	
Black	3221 (25.7)	1,879 (22.7)	1,342 (31.5)	
Other	880 (7.0)	600 (7.25)	280 (6.6)	
Married, *n* (%)	6,384 (50.1)	4,367 (51.9)	2,017 (46.5)	**<0.001**
English preferred language, *n* (%)	12,426 (97.4)	8,197 (97.4)	4,229 (97.5)	0.54
Active patient portal use^a^, *n* (%)	10,151 (79.6)	6,820 (81.0)	3,331 (76.8)	**<0.001**
Patient health conditions^b^, *n* (%)				
Hypertension	10,221 (80.1)	6,710 (79.7)	3,511 (80.9)	0.09
Rheumatoid arthritis/osteoarthritis	4,539 (35.6)	3,031 (36)	1,508 (34.8)	0.17
Diabetes	4,266 (33.4)	2,734 (32.5)	1,532 (35.3)	**<0.001**
Chronic kidney disease	3,300 (25.9)	2,059 (24.5)	1,241 (28.6)	**<0.001**
Chronic obstructive pulmonary disease	2,574 (20.2)	1,663 (19.8)	911 (21.0)	0.09
Hearing loss	2,431 (19.1)	1,537 (18.3)	894 (20.6)	**<0.001**
Dementia	915 (7.2)	473 (5.6)	442 (10.2)	**<0.001**

*Note*. Twelve-month observation period, October 3, 2021 to October 2, 2022.

^a^Active patient portal use defined as logging in to the portal and completing at least one activity in the last year.

^b^ICD codes or problem list as of October 2021, ICD codes included identified via Chronic Conditions Data Warehouse algorithm (30).

^c^Bold indicates significant at the α = .05 level.

Compared with older adults who did not incur a billed AWV, those who did were more likely to be White (70.0% vs 62.0%; *p* < .001) or married (51.9% vs 46.5%; *p* < .001). Black older adults were less likely to incur a billed AWV (22.7% vs 31.5%; *p* < .001). Older adults who were active portal users were more likely to incur a billed AWV than those who were not, though active portal use was high in both groups (81.0% billed AWV vs 76.8% not billed; *p* < .001). Those with a billed AWV (compared to without) were more likely to have a documented diagnosis of diabetes, chronic kidney disease, hearing loss, or dementia. No clinically meaningful difference was observed by age, or by hypertension, chronic obstructive pulmonary disease, or rheumatoid arthritis/osteoarthritis diagnosis.

### Older Adult Responses to the AWV Health Risk Assessment

Among older adults with a billed AWV, the 5 most frequently affirmative health risk assessment categories were: seeing other providers (70.6%), falls or balance concerns (44.2%), lack of a documented advanced directive (42.3%), sedentary behaviors (39.9%), and incontinence (35.1%) (Table 2). Affirmative responses to health risk assessment categories differed by active portal status for 11 of 17 social/mental health risk categories and 7 of 11 physical health risk categories.

**Table 2.** Medicare AWV Affirmative Responses to Health Risk Assessment Categories by Portal User Status

Health risk assessment category	Overall		Active portal user^a^ *n* = 6820		Nonportal user^a^ *n* = 1600		Percent difference^b^
	Answered (*n*)	Affirmative response (*n*, %)	Answered (*n*)	Affirmative response (*n*, %)	Answered (*n*)	Affirmative response (*n*, %)	%
Mental health							
Seen other providers	7978	5637, 70.7	6458	4767, 73.9	1520	870, 57.2	16.6
Sedentary/low exercise	7705	3054, 39.6	6261	2349, 37.5	1444	705, 48.8	−11.3
No advance directive	7341	2740, 37.3	6001	2109, 35.1	1340	631, 47.1	−12.0
No dentist in last year	7954	2845, 35.8	6454	2017, 31.3	1500	828, 55.2	−23.9
Depression	8238	2366, 28.7	6677	1896, 28.4	1561	470, 30.1	−1.7
Live alone	7847	2102, 26.8	6368	1554, 24.4	1479	548, 37.1	−12.7
Vitamins/supplement	7924	1745, 22.0	6431	1456, 22.6	1493	289, 19.4	3.2
Alcohol	7921	1275, 16.1	6436	1136, 17.7	1485	137, 9.2	8.5
Poor diet	7840	1356, 17.3	6371	1076, 16.9	1469	280, 19.1	−2.2
Money/medication issues	8040	937, 11.7	6511	740, 11.4	1529	197, 12.9	−1.5
Caregiver	7841	882, 11.3	6374	719, 11.3	1467	163, 11.1	0.2
Transportation needs	8069	653, 8.1	6529	446, 6.8	1540	207, 13.4	−6.6
Smoking	7808	487, 6.2	6358	326, 5.1	1450	161, 11.1	−6.0
Unsafe at home	7929	163, 2.1	6446	123, 1.9	1483	40, 2.7	−0.8
Drugs	7906	140, 1.8	6425	114, 1.8	1481	26, 1.8	0.0
Abuse	7805	126, 1.6	6350	102, 1.6	1455	24, 1.7	−0.1
Lack of seatbelt use	7943	114, 1.4	6446	70, 1.1	1497	44, 2.9	−1.8
Physical health							
Falls/balance	8245	3655, 44.3	6676	2914, 43.7	1569	741, 47.2	−3.5
Incontinence	8062	2839, 35.2	6525	2285, 35.0	1537	554, 36.0	−1.0
Mobility	8014	2688, 33.5	6481	2029, 31.3	1533	659, 43.0	−11.7
Pain/fatigue	8109	2424, 29.9	6562	1922, 29.2	1547	502, 32.5	−3.3
Memory concerns	8044	1713, 21.3	6508	1375, 21.1	1536	338, 22.0	−0.9
Housework needs	8032	1643, 20.5	6498	1248, 19.2	1534	395, 25.8	−6.6
Hearing concerns	8006	1485, 18.6	6480	1168, 18.0	1526	317, 20.8	−2.8
Cognition concerns	8030	1096, 13.7	6501	886, 13.6	1529	210, 13.7	−0.1
ADL difficulty	8025	906, 11.3	6493	686, 10.6	1532	220, 14.4	−3.8
Vision concerns	7974	668, 8.4	6459	477, 7.4	1515	191, 12.6	−5.2
Poor self-reported health	8075	439, 5.4	6540	338, 5.2	1535	98, 6.4	−1.2

*Note*s. ADL = activities of daily living. Twelve-month observation period, October 3, 2021 to October 2, 2022.

^a^Active portal use defined as having activated a portal account, logging in and completing at least 1 task during the year. Nonportal user was defined as someone who has not completed a portal activity during the year of study.

^b^Absolute difference (active user minus nonportal user).

The magnitude and directionality of absolute between-group differences by portal use status visualized from largest to smallest percent difference (relative and absolute) as presented in Supplementary Figure 1. Compared with nonactive portal users, active portal user responses suggest healthier behaviors, less dependency, and greater engagement in preventive behaviors: all but 4 risk categories (seeing other providers, alcohol use, taking vitamins/supplements, and caregiving) were affirmative more often by nonportal users. Seven of the 8 largest absolute between-group differences were observed for social/mental health risk categories, which were affirmative more often by nonportal users. Active portal users were more likely to report seeing a dentist (23.9% difference), living alone (12.7% difference), and were less likely to report mobility concerns (11.7% difference), sedentary behavior (11.1% difference), and need for housework or transportation support (6.6% and 6.6% difference, respectively) than nonportal users.

### Health Risk Assessment Category and Patient Portal Messaging

**Figure 1.**
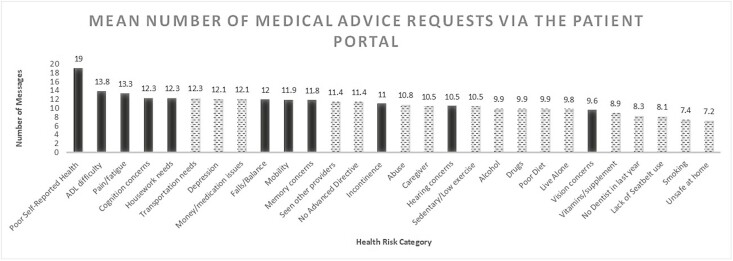
Mean number of patient-initiated secure portal messages. Mean of an individual participant’s mean number of messages among active portal users who had an AWV; 12-month observation period, October 3, 2021 to October 2, 2022; dashed = social and/or mental health risk, solid = physical health risk. ADL = activities of daily living; AWV = Annual Wellness Visit.

**Figure 2.**
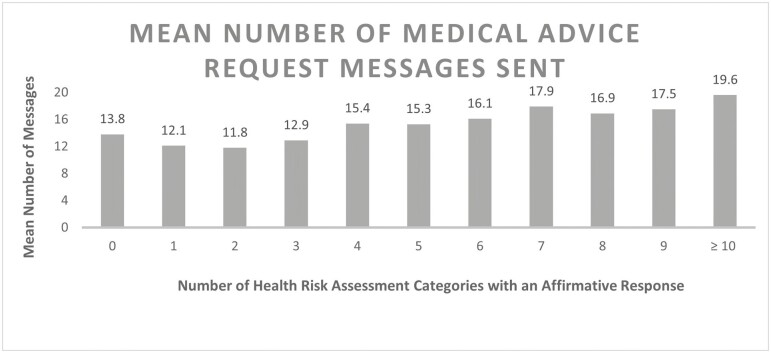
Mean number of patient-initiated portal messages by number of health risk assessment concerns. Twelve-month observation period, October 3, 2021 to October 2, 2022.

Mean number of patient-initiated portal messages for medical advice ranged from 7.2 to 19.0 by affirmatively answered health risk categories (Figure 1). Patient-initiated portal messages were greatest among patients who affirmatively answered poor self-rated health, activities of daily living difficulty, pain/fatigue, cognition concerns, and housework needs on their health risk assessment. The mean number of messages was incrementally greater with greater number of affirmative risk categories (Figure 2), fluctuating from 13.8 for those with no affirmatively answered categories, to 19.6 for those with 10 or more affirmatively answered categories.

## Discussion

This study draws on electronic medical record data from a large integrated academic health system to assess opportunities to facilitate portal-based administration of the Medicare Annual Wellness Visit. We found that 8 in 10 older adults eligible for an AWV were active patient portal users, and that patient portal users were slightly more likely than nonportal users to incur a billed AWV. Compared with nonportal users, active portal users affirmatively answered fewer preventive care health concerns and were more likely to report engaging in protective health behaviors. We also observed variability in the volume of patient-initiated portal messages for medical advice by affirmatively answered health risk categories. A greater volume of messages during the year of observation was observed among those who answered affirmatively to risks within the physical (vs mental) health domain, and those who answered affirmatively to greater numbers of risk categories. Taken together, findings indicate that portal-based administration of the AWV health risk assessment is more likely to reach a healthier and more literate population of older adults (32), although implementation and potential increased reliance on the patient portal must carefully attend to and evaluate the implications for key patient populations of interest in relation to accessibility and equity.

Previous studies that have examined AWV completion have defined visits using billing codes (24,33), without capturing specific health risk concerns (15). Our study is one of the few (32) to examine specific patient-reported concerns from the full health risk assessment. We find that the most commonly affirmative concerns relate to falls and/or balance and incontinence within the physical health domain, the absence of an advanced directive, and presence of sedentary behavior for the mental health domain. Affirmatively answered categories of the health risk assessments differed by portal use with 24 of 28 categories more commonly affirmative by nonportal users. Our study highlights opportunities for portal-based intervention (eg, print and/or video educational materials and tools, availability of referrals, prescripted medical advice message templates) for frequently affirmative health risks among active portal users of mobility concerns, living alone, sedentary behaviors—but that risk prevention efforts must involve multiple modalities to meet variable preferences and capacity for information sharing and learning.

This analysis of patient portal use associated with the AWV is an example of the vision for care delivery and research articulated in the learning health system (1,34,35). This vision aligns the patient voice with research and clinical care and integrates internal health system data with patient experiences (35–38). The additional time commitment by staff to administer the AWV has been identified as a barrier to incorporating patient health risk assessments into routine care, impeding facilitation of a reimbursable and structured platform to integrate patient-identified health risks into care (21,23). The ability to integrate the health risk assessment with digital health technology aligns with principles of the learning health system, but also has the potential to reduce workflow needs or tasks for risk assessment and AWV preventive care administration. Risk assessment completion and nonresponse as documented in the medical record appears to be differential by portal use status in our data sample, with nonusers of the portal having lower response completion and higher frequency of affirmative responses to risk categories than portal users. This potentially presents a selection bias for some health risk assessment categories, leading to an underestimate of risk in our study. It is reasonable to consider patients who are active portal users may be more active participants in their healthcare process and therefore may be partners in ensuring health risk assessment completion. Our study is one of the first to incorporate direct risk assessment responses into investigation of the AWV. Considerations of such are warranted when understanding enactment of patient portal facilitation of AWV components in alignment with advocation of portal registration initiatives.

The AWV is a complex visit that affords reimbursement opportunities to enable the collection of patient-reported information and a comprehensive consultation (39), setting the stage for more effective collaboration and care decision-making (40). We found the volume of patient-initiated medical advice messages was incrementally greater with numbers of affirmatively answered AWV health risks, with noteworthy differences in volume across type of identified health risk. It is notable that concerns often unique to older adults such as pain/fatigue, cognition, and the ability to complete activities of daily living received the largest volume of medical advice requests but were areas with less distinct difference in reporting by portal use status. Expanded access to the health risk assessment through embedding responses directly in the patient portal has the potential to preemptively initiate integrated patient education, proactively address health concerns for differing stages of prevention, stimulate referrals, reduce personnel demands, and decrease demands for care on provider’s time (3,38,41).

Our study is one of the few to integrate patient responses with billed evidence of AWV completion from a large and diverse academic health system. Study limitations include investigation within a single health system, our organization’s update to the health risk assessment form during the observation period, and minimal completion of the health risk assessment via the patient portal. Our observation period incorporates dates around the COVID-19 pandemic and the associated changes to portal use which have been observed and sustained more broadly. Study findings inform how health systems may approach informed AWV facilitation through the patient portal; however, our study incorporates a single health system so may not be generalizable to portal-based practices in other regions. Our study was limited in exploration of sociodemographic factors that may influence both portal use and AWV completion due to limitations in quality of patient measures recorded in the electronic medical record. Although we observed lower AWV completion for patients with specific chronic conditions such as hearing loss or dementia, prior work has demonstrated decreased healthcare utilization or lack of regular source of care for each of these conditions, possibly related to communication barriers (42,43). Our findings align with other studies which suggest interventions through the AWV may be inclusive of healthier older adults or a population that is more care-seeking which should be considered when designing future interventions or defining study outcomes (19,21,44). Although embedding the AWV health risk assessment could streamline clinic administration, such efforts must attend to equity issues and the need to support multiple modalities.

## Supplementary Material

igae023_suppl_Supplementary_Table_S1_Figure_S1

## Data Availability

Given the identifiability of the data shared in this manuscript, the data are not available. The analytic methods are available to researchers upon reasonable request. Researchers should email kgleaso2@jhu.edu to request the analytic methods. The studies reported in the manuscript were not preregistered.
