# Supplementary material for: The Annual Wellness Visit Health Risk Assessment: Potential of Patient Portal-Based Completion and Patient-Oriented Education and Support
Source: Innov Aging. 2024 Feb 29;8(3):igae023. doi: 10.1093/geroni/igae023 (PMC11010311; doi:10.1093/geroni/igae023)
Supplement: igae023_suppl_Supplementary_Table_S1_Figure_S1 [file igae023_suppl_supplementary_table_s1_figure_s1.docx]

*Innovation in Aging* Supplementary Material: Danielle S. Powell, Mingche M. J. Wu, Stephanie Nothelle, Kelly Gleason, Esther Oh, Hillary D. Lum, Nicholas S. Reed, & Jennifer L. Wolff. The Annual Wellness Visit Health Risk Assessment: Potential of Patient Portal-Based Completion and Patient-Oriented Education and Support.

**Supplementary Table 1**. Medicare Annual Wellness visit health risk assessment form reconciliation and health risk categorization for a large academic medical center (October 3, 2021-October 2, 2022)

| **Health Risk Assessment Category** | **PROMIS domain** | **Old Health Risk Assessment Form** | **New Health Risk Assessment Form** |
| --- | --- | --- | --- |
| **Self-Reported Health** | Social and/or mental health | How does your health compare to most people your age? | How does your health compare to most people your age? |
| **ADL Difficulty** | Physical health | Do you have trouble dressing, bathing, eating, using the toilet, or grooming? | Do you have trouble dressing, bathing, eating, using the toilet, or grooming? |
| **Transportation Needs** | Social and/or mental health | Do you have trouble doing errands alone such as visiting your doctor or shopping? | In the past year, has lack of transportation kept you from medical appointment or from getting medications? |
|  |  |  | In the past year, has lack of transportation kept you from meetings, work, or getting things needed for daily living? |
| **Housework needs** | Social and/or mental health | Do you have trouble making food, doing housework, using the phone, or transportation? | Do you need help making food or doing housework? |
| **Money/medication issues** | Social and/or mental health | Do you have trouble using your checkbook, paying bills, or taking medicine? | Do you have trouble managing your money or medications? |
| **Incontinence** | Physical health | Do you leak urine or soil your under clothes? | Do you leak urine? |
|  |  |  | Do you leak stool or lose control of your bowels? |
| **Mobility** | Physical health | Do you have serious difficulty walking or climbing stairs? | Do you have serious difficulty walking or climbing stairs? |
| **Falls/Balance** | Physical health | Have you tripped or fallen during the last year? | Have you tripped or fallen during the last year? |
|  |  | Do you have trouble keeping your balance? | Do you have trouble keeping your balance? |
| **Hearing Concerns** | Physical health | Are you deaf or do you have serious trouble hearing? | Are you deaf or do you have serious trouble hearing? |
| **Vision concerns** | Physical health | Are you legally blind or do you have serious trouble seeing, even if you wear glasses? | Are you legally blind or do you have serious trouble seeing, even if you wear glasses? |
| **Sedentary/Low exercise** | Social and/or mental health | Do you exercise on a regular basis? | On average, how many days per week do you engage in moderate to strenuous exercise (like a brisk walk)? |
| **Depression** | Social and/or mental health | Have you felt unusual stress, anger, or loneliness in the last 14 days? | Over the last 2 weeks, how often have you been bothered by feeling down, depressed, or hopeless? |
|  |  | Have you felt depressed, down or hopeless in the last 14 days? | Over the last 2 weeks, how often have you been bothered by little interest or pleasure in doing things? |
|  |  | Have you lost pleasure in doing things you enjoy in the last 14 days? |  |
| **Abuse** | Social and/or mental health | Are you a victim of physical, sexual, or emotional abuse? | Within the last year, have you been humiliated or emotionally abused in other ways by our partner or ex-partner? |
|  |  |  | Within the last year, have you been kicked, hit, slapped, or otherwise physically hurt by your partner or ex-partner? |
|  |  |  | Within the last year, have you been afraid of yoru partner or ex-partner? |
| **Pain/Fatigue** | Physical health | have you felt unusual pain or fatigue in the last 14 days? | In the past 2 weeks, have you had more pain than ususal? |
|  |  |  | In the past 2 weeks, have you had more fatigue than usual? |
| **Caregiver** | Social and/or mental health | If you live with someone, is that person in good health? | Are you a caregiver for another person? |
|  |  | Whom do you live with? | Whom do you live with? |
| **Alcohol** | Social and/or mental health | Do you drink alcohol, use tobacco, or take illicit drugs? | How often do you have a drink containing alcohol? |
|  |  |  | How many drinks containing alcohol do you have on a typical day when you are drinking? |
| **Drugs** | Social and/or mental health |  | Do you use recreational drugs or prescription medications for non-medical reasons? |
| **Smoking** | Social and/or mental health |  | Tobacco use |
| **Poor diet** | Social and/or mental health | Do you frequently eat fruits, vegetables, fiber and whole grains? | Do you think you diet is unhealthy? |
|  |  | Do you frequently use sugar, salt or eat fatty or fried foods? |  |
| **Vitamins/supplements** | Social and/or mental health | Do you take calcium or vitamin supplements? | Do you take herbal or vitamin supplements that are not on your medication list? |
| **No dentist in last year** | Social and/or mental health | Have you seen a dentist during the last year? | Have you been to a dentist during the last year? |
| **Lack of Seat belt use** | Social and/or mental health | Do you use a seat belt when riding in a vehicle? | Do you use a seat belt when riding in a vehicle? |
| **Unsafe feeling at home** | Social and/or mental health | Do you have any safety concerns at home? | Do you feel safe at home? |
| **Memory concerns** | Physical health | Are you or is someone close to you concerned about your memory? | Do you have memory problems in the past year? |
| **Cognition concerns** | Physical health | Do you have serious trouble concentrating on things, remembering things, or making decisions? | Do you have trouble thinking clearly or making decisions? |
| **Seen other Providers** | Social and/or mental health | Do you have other providers that give you care on a regular basis? | Have you seen other providers in the past year? |
| **No Advance Directive** | Social and/or mental health | Do you have a medical power of attorney/advance directive? | Do you have a medical power of attorney/advance directive? |

**Supplementary Figure 1.** Difference in Affirmative Responses to Health Risk Assessment Categories by Portal User Status

**A**

**B**

Notes: A) Absolute difference (Active user – non-portal user); B) Relative difference (relative difference/ average of active portal user positive + non-portal user positive response). A relative percent difference (active portal users minus non-portal users) places the difference in health risk affirmative responses within the context of public health utility given differences in the overall number or active portal users to non-users in our sample. This difference presents the denominators as the underlying sample who has completed and then answered affirmatively to the risk— a small percent difference among a large denominator may have greater public health utility than a large percent difference among a small denominator; dashed = social and/or mental health risk, solid = physical health risk

*indicates signficant difference in health risk by Portal activity (α=0.05)
